# iTRAQ-Based Quantitative Proteomic Analysis of Embryogenic and Non-embryogenic Calli Derived from a Maize (*Zea mays* L.) Inbred Line Y423

**DOI:** 10.3390/ijms19124004

**Published:** 2018-12-12

**Authors:** Beibei Liu, Xiaohui Shan, Ying Wu, Shengzhong Su, Shipeng Li, Hongkui Liu, Junyou Han, Yaping Yuan

**Affiliations:** College of Plant Science, Jilin University, Changchun 130062, China; liubeibei1985@126.com (B.L.); shanxiaohui@jlu.edu.cn (X.S.); wuying@jlu.edu.cn (Y.W.); sushengzhong@jlu.edu.cn (S.S.); lisp@jlu.edu.cn (S.L.); liuhk@jlu.edu.cn (H.L.); hanjy@jlu.edu.cn (J.H.)

**Keywords:** iTRAQ, proteomics, somatic embryogenesis, pyruvate biosynthesis, *Zea mays*

## Abstract

Somatic embryos (SE) have potential to rapidly form a whole plant. Generally, SE is thought to be derived from embryogenic calli (EC). However, in maize, not only embryogenic calli (EC, can generate SE) but also nonembryogenic calli (NEC, can’t generate SE) can be induced from immature embryos. In order to understand the differences between EC and NEC and the mechanism of EC, which can easily form SE in maize, differential abundance protein species (DAPS) of EC and NEC from the maize inbred line Y423 were identified by using the isobaric tags for relative and absolute quantification (iTRAQ) proteomic technology. We identified 632 DAPS in EC compared with NEC. The results of bioinformatics analysis showed that EC development might be related to accumulation of pyruvate caused by the DAPS detected in some pathways, such as starch and sucrose metabolism, glycolysis/gluconeogenesis, tricarboxylic acid (TCA) cycle, fatty acid metabolism and phenylpropanoid biosynthesis. Based on the differentially accumulated proteins in EC and NEC, a series of DAPS related with pyruvate biosynthesis and suppression of acetyl-CoA might be responsible for the differences between EC and NEC cells. Furthermore, we speculate that the decreased abundance of enzymes/proteins involved in phenylpropanoid biosynthesis pathway in the EC cells results in reducing of lignin substances, which might affect the maize callus morphology.

## 1. Introduction

Maize is one of the most important cereal crops in the world. Maize production is directly related to the agricultural economy and farmers’ income [1]. With the development of molecular breeding and transgenic technology, more and more new genetically modified (GM) maize varieties have been developed and widely cultivated, which has made a tremendous contribution to raising farmers’ incomes and ensuring food security (http://www.isaaa.org/resources/publications/briefs/). However, many problems still limit the development of GM maize. For example, because of the majority of maize genotypes showing low embryogenic growth response in culture, only several inbred lines can be used for maize genetic transformation, such as A188 which displays a high embryogenic culture response [2,3,4]. Somatic embryos (SE), which have the potential to rapidly form a whole plant, have been widely used to propagate transgenic organisms and to obtain genetically modified plants [5]. Efficient SE production also has significance for many agricultural biotechnology applications such as clonal propagation, production of synthetic seed [6], and gamete cycling in rapid breeding [7]. Understanding the mechanism of maize SE development is a key issue to be solved [5]. The first issue is to find the key regulatory pathways that could induce the development of embryogenic callus (EC, can generate SE) rather than nonembryogenic callus (NEC, can’t generate SE) during somatic embryogenesis.

A great deal of differentially expressing genes were identified to be related with EC development in maize by cDNA-AFLP and RNA-seq [8,9], but the mRNA levels usually do not correlate well with the protein abundances and functions, due to various post-translational modifications. Unlike RNA, proteins are the central biomolecules that are responsible for all cellular functions in the living organism. Therefore, the analysis of the differential proteins between EC and NEC can more accurately explore the key factors affecting somatic embryogenesis. Early studies have found that many extracellular proteins can affect somatic embryogenesis, in which arabinogalactan protein (AGPs), nonspecific lipid transfer protein (nsLTPs) and germin-like proteins (GLPs) had been considered as marker proteins in somatic embryogenesis. AGPs were reported that played a facilitating role during the process of embryo development or somatic embryogenesis in maize [10], wheat [11] and carrot [12]. And the specific expression of GLPs was detected during the early development of somatic embryos in *Pinus caribaea* Morelet [13]. In recent years, high-throughput proteomic technology was successfully used to understand the process of somatic embryogenesis in different plants. In *Medicago truncatula*, 136 differentially abundant proteins were detected between embryogenic and nonembryogenic lines [14]. Additionally, the reference maps with 169 proteins were established for embryogenic cell lines delivered from *M. truncatula* protoplasts [15]. Four glycolytic enzymes, namely uracil-diphosphate (UDP)-glucose pyrophosphorylase, fructose bisphosphate aldolase, triosephosphate isomerase, and glyceraldehyde-3-phosphate dehydrogenase were found up-accumulated in somatic embryos of *Cyclamen persicum* [16]. Related study also suggested that the proteins related with auxin releasing might be important for late developmental stages of somatic embryos [17]. Two-dimensional electrophoresis (2-DE) was performed to compare the proteomes of embryogenic and nonembryogenic calli induced from H99 inbred maize line. Some proteins associated with somatic embryogenesis were enriched in cell proliferation, transcription and protein processing, stress response, signal transduction, metabolism and energy pathways [18]. And in the grape (*Vitis vinifera*) [19], orange (*Citrus sinensis* Osbeck) [20], oil palm (*Elaeis guineensis* Jacq.) [21], the proteome changes during somatic embryogenesis were also studied by 2-DE technique. The study results showed that the differentially accumulated proteins were mostly related to carbohydrate and energy metabolism or oxidative stress. At the same time, a large number of unknown proteins have also been found and need to be further explored. Isobaric tags for relative and absolute quantification (iTRAQ) is a new proteome research method which can identify low-abundance protein species and the protein species that are too large/small, too acidic/basic and too hydrophobic to be detected by 2-DE [22,23]. Recently, iTRAQ has been used to explore the proteome of somatic embryogenesis in *Gossypium hirsutum* L. [24], *Larix principis-rupprechtii* Mayr [25]. Although these studies have found some proteins associated with somatic embryogenesis, the results cannot systematically elucidate the mechanism that affects the development of EC and NEC in the process of somatic embryogenesis, and which proteins could promote calli to transform into EC or SE. 

As reported before, we have identified a new receptor of an elite maize (*Z. mays* L.) inbred line for genetic transformation which displaying high efficiency via intact somatic embryogenesis [8,9]. Therefore, the purpose of this study is to compare the proteomes between EC and NEC by iTRAQ, and then to analyze the differential proteins to explore key proteins or metabolic pathways that affect the development of EC or NEC. This will provide a theoretical basis or working model for solving the problem of EC induction in somatic embryogenesis.

## 2. Results

### 2.1. Induction of Embryogenic and Nonembryogenic Calli

**Figure 1:**
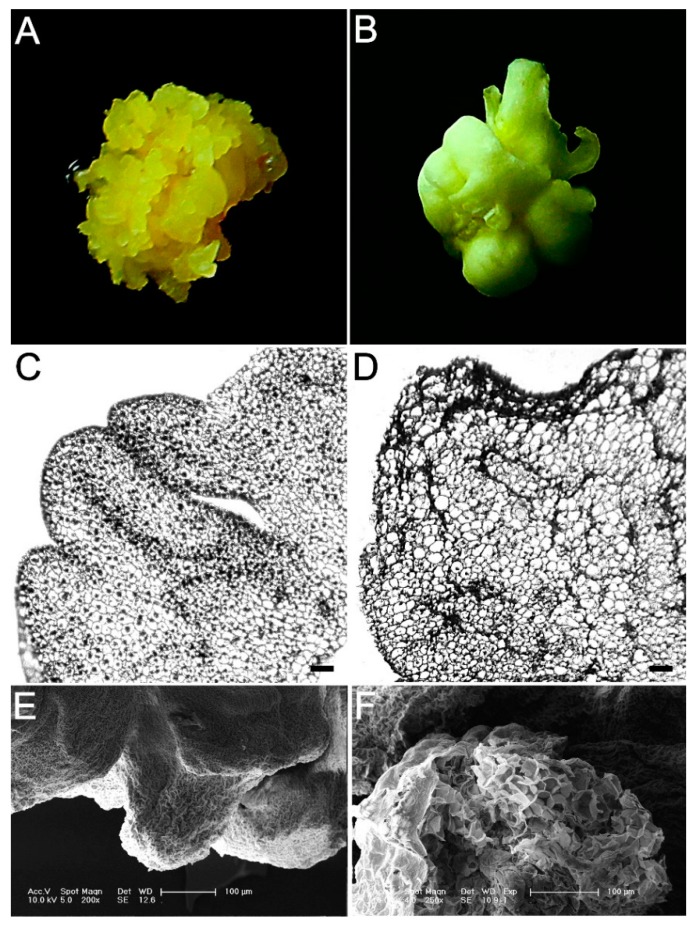
Morphological and histological analysis of embryogenic calli (EC) and nonembryogenic calli (NEC). (**A**) Morphological analysis of EC; (**B**) morphological analysis of NEC; (**C**) histological analysis of EC (Bar: 200 μm); (**D**) histological analysis of EC (Bar: 200 μm); (**E**) scanning electron microscope analysis of EC (Bar: 100 μm); (**F**) scanning electron microscope analysis of NEC (Bar: 100 μm).

Somatic embryo (SE) offers great potential in plant propagation but its developmental process is so complex. In order to induce somatic embryos, immature embryos of inbred maize line Y423 were placed on the induction media. After induction and subculturing 4–5 times, globular somatic embryos that were yellow, loose and small granular appeared on the surfaces of EC. In contrast, NEC looked pale yellow and compact (Figure 1A,B). Histological analysis revealed that the EC cells were clusters of cells with large nuclei and dense cytoplasm (Figure 1C), whereas the NEC cells had vacuoles and few plastids with a loose cell arrangement without rules (Figure 1D). The scanning electron microscope (SEM) analysis of the epidermal cells revealed that the EC had granule structures on their rough surface (Figure 1E), but NEC had a smooth and flaky surface structure (Figure 1F). All these showed that there was obvious difference at both morphological and cellular levels between EC and NEC.

### 2.2. Primary Data Analysis and Protein Identification

To investigate the differences during maize SE development on protein perspective, proteins of EC and NEC induced from Y423 were extracted and analyzed using an iTRAQ-based shotgun proteomics strategy. Samples were double labeled with iTRAQ tags for higher confidence in identification. Totally 286,622 spectra were generated after three biological replicates data merging from. Mascot identified a total of 39,493 spectra matched to known spectra, 28,830 spectra matched to unique spectra, 19,842 peptides, 15,878 unique peptides and 5592 proteins. The peptide length distribution, the peptide number distribution, protein mass distribution, and distribution of protein’s sequences coverage were provided in Supplementary Figure S1A–D, respectively. Around 57% of the proteins included at least two peptides. Protein sequences coverage with 40–100%, 30–40%, 20–30%, 10–20%, and under 10% variation accounted for 5%, 6%, 14%, 24%, 51%, respectively. 

### 2.3. Identification of Differentially Accumulated Protein Species (DAPS) by iTRAQ

A protein species was considered differentially accumulated as it exhibited a fold change > 1.2 and a *p*-value < 0.05 with a false discovery rate (FDR) of <5%. Based on these two criteria, 632 DAPS were identified, of which, 366 were up-accumulated and 266 were down-accumulated in EC compared with NEC. Supplementary Table S2 shows the DAPS detailed information.

### 2.4. Bioinformatics Analysis of DAPS Identified by iTRAQ

**Figure 2:**
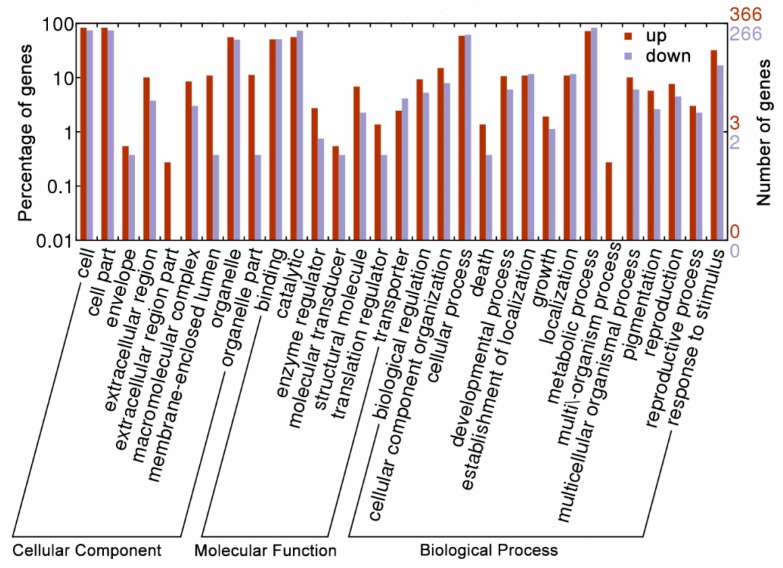
Gene ontology (GO) annotation of the differential abundance protein species (DAPS) in EC compared to NEC. The up-accumulation means a higher protein relative abundance in EC than in NEC, and the down-accumulation means a lower protein relative abundance in EC than in NEC.

To identify the significantly enriched GO functional groups of DAPS, GO annotation was carried out. The DAPS between EC and NEC were classified into 31 functional groups (Figure 2), of which biological processes accounted for 15 GO terms (the most representative were “metabolic processes”), cellular components accounted for 9 GO terms (the most representative were “cell” and “cell part”), and molecular functions accounted for 7 GO terms (the most representative was “catalytic”). The results also showed that some DAPS clustered into the groups of extracellular region part and multi\-organism process just including the up-accumulated DAPS in EC. In addition, the quantity of up-accumulated DAPS was obviously higher than that of down-accumulated in the groups of membrane-enclosed lumen and organelle part. In contrary, the quantity of up-accumulated DAPS was lower than that of down-accumulated ones in the groups of “catalytic”, “transporter”, and “metabolic process”.

**Figure 3:**
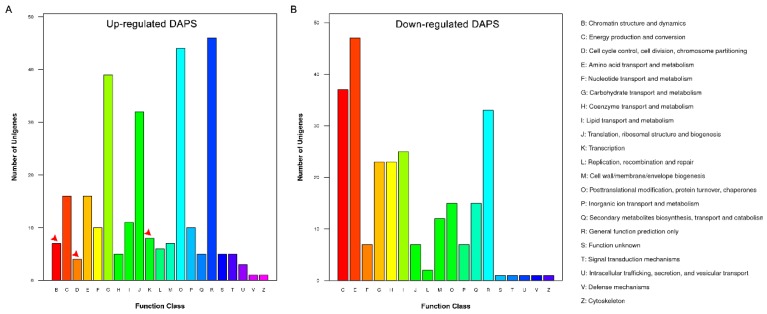
Clusters of orthologous groups of proteins (COG) classification of differential abundance protein species (DAPS) in EC and NEC. The arrows mark the groups just found in the upregulated DAPS. (**A**) Up-regulated DAPS; (**B**) Down-regulated DAPS.

A total of 539 DAPS (85.3%) between EC and NEC identified by iTRAQ were classified into 21 clusters of orthologous groups of proteins (COG) categories. In up-accumulated DAPS, “general function prediction only” represented the largest group, followed by “post-translational modification”, “protein turnover, chaperones”, “carbohydrate transport and metabolism”, and “translation, ribosomal structure and biogenesis” (Figure 3A). In down-accumulated DAPS, “amino acid transport and metabolism” represented the largest group, followed by “energy production and conversion”, “general function prediction only” and “lipid transport and metabolism” (Figure 3B). Especially, three groups, “chromatin structure and dynamics” (8 DAPS), “cell cycle control, cell division, chromosome partitioning” (4 DAPS) and “transcription” (8 DAPS), were just found in the classification of up-accumulated DAPS (Figure 3, Table 1). These DAPS might be the key proteins during the development of EC in maize. 

### 2.5. Transcriptional Analysis of Selected Genes for the Differentially Expressed Proteins

**Figure 4:**
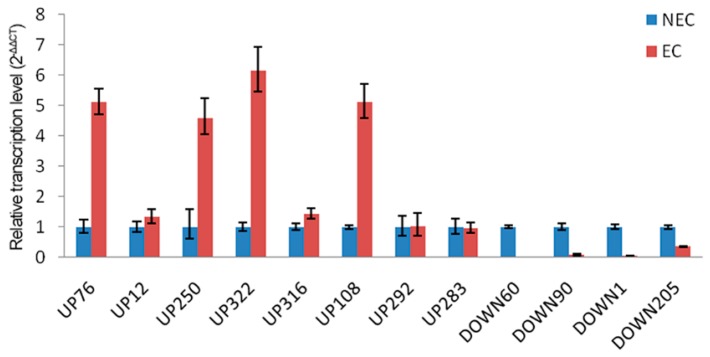
Analysis of transcript levels of the differential abundance protein species between EC and NEC by qRT-PCR.

To elucidate the correspondence between the mRNA and protein level and confirm the authenticity and accuracy of the proteomic analysis, transcriptional analysis of 12 protein species, eight up-accumulated DAPS and four down-accumulated DAPS, was performed by qRT-PCR (Figure 4, Table 2). Of the selected proteins, 10 genes displayed accordant change tendency on the transcript level as the results of iTRAQ, such as nucleotide pyrophosphatase, putative subtilase family protein, peroxidase superfamily protein and terpenoid cyclases. By contrast, two genes (rhicadhesin receptor precursor and C cell wall invertase 2) showed no significant difference on the transcription level despite the detection of differential expression patterns on the protein level. The discrepancy between the transcription level of the two genes and the abundance of the corresponding protein species probably resulted from various posttranslational modifications between EC and NEC, such as protein phosphorylation and glycosylation.

## 3. Discussion

The first report about the use of somatic embryogenesis for maize tissue culture was published in 1975 [26]. However, it is very difficult to obtain somatic embryos from most of maize elite inbred lines because of the genetic diversity of maize. Well-known receptors for maize genetic transformation, just including A188, HiII, H99, and some other inbred lines, can been inducted to embryonic calli with high efficiency [9,27,28]. Nevertheless, many of these inbred lines were not suitable for productive applications because of their weak combining ability. A new-screened elite inbred line, Y423, can be inducted easily to develop somatic embryos and can be used in both production and scientific research [8]. However, NEC is obtained with EC at the same time during the process of SE induction, which will increase costs and is unfavorable to GM maize production. Therefore, it is necessary to study the mechanism of EC and NEC development. 

Protein is substance basis of life and specific practitioner of life activities. Although transcriptome analysis is very useful to reveal the differential expression between EC and NEC, the expression level of mRNA is not a good predictor of the abundance of the corresponding proteins due to several factors such as post-translational modifications. Therefore, research on proteomics is helpful to reveal complex difference between EC and NEC in maize and provides new information concerning EC development. iTRAQ was explored for proteomics profiling and enrichment of differentially expressed analysis between EC and NEC of the inbred maize line Y423. As a result, 632 DAPS were identified, among which, 366 DAPS were up-accumulated and 266 DAPS down-accumulated proteins detected in EC compared with NEC. Bioinformatics analysis showed that DAPS were annotated in 31 GO functional groups, 539 DAPS were classified into 21 COG categories, and 512 DAPS were identified to 106 KEGG pathways. Some significant DAPS were grouped into categories based on the GO, COG, and KEGG analysis which we discussed below.

In order to elucidate the functional difference of DAPS between EC and NEC, we analyzed the COG of high and low abundance proteins in EC, respectively. The results showed 20 special up-accumulated DAPS in EC versus NEC were clustered in three classifications (“chromatin structure and dynamics”, “cell cycle control, cell division, chromosome partitioning” and “transcription”) (Table 1). Most of the DAPS, in the classification of “chromatin structure and dynamics”, were histones which are basic proteins, rich in basic amino acids such as arginine and lysine. Some studies have shown that high level of arginine was detected in embryogenic callus, which is beneficial to cell division and cell differentiation [29]. Arginine, as an important component, was also added to the medium in cotton somatic embryo tissue culture [30,31,32]. In addition, some studies have shown calmodulin, in the classification of “cell cycle control, cell division, chromosome partitioning”, played a very important role in somatic embryogenesis. In carrot somatic embryos, calmodulin was localized in the shoot apical meristem region and was strongly expressed in the globular and cardiac phases, whereas it was less expressed in undifferentiated calli [33]. During the somatic embryogenesis of sugarcane, calmodulin accelerated cell differentiation, and the content of calmodulin was higher at any stage of somatic embryo as compared to undifferentiated calli [34].

To study which metabolic pathway plays a major role in the induction of EC, KEGG pathway enrichment analysis was explore. 512 DAPS were mapped in 106 KEGG pathways, in which pyruvate metabolism not only was the most significantly enriched pathway of DAPS, but also the number of enriched DAPS is up to 25. In starch and sucrose metabolism and glycolysis/gluconeogenesis closely related to pyruvate metabolism, a large number of DAPS was also enriched significantly, reaching 24 and 23, respectively (Table S3). We also noted that more than 20 DAPS were enriched in the ribosomes and the phenylpropanoid biosynthesis pathways. 

Thirty DAPS were detected in ribosomal pathway, accounting for 6% of total differential proteins, of which 25 DAPS were up-accumulated and only five were down-accumulated in EC (Figure S2, Table 3). Most of up-accumulated DAPS were clustered in the classification of “translation”, “ribosomal structure and biogenesis”, which was consisted with the COG results of up-accumulated DAPS. These DAPS are mainly related to the 40S small subunit and 60S large subunit (Table 3). This result may indicate that protein synthesis in EC was more exuberant than that in NEC. Ribosome-inactivating protein, which inhibits ribosomal formation, was not detected in the EC of spinach (*Spinacia oleracea*) [35]. This also illustrated that reducing the abundance of proteins, which inhibit the formation of ribosomes in EC, might result in a significant increase in the number of ribosomes which in turn would promote protein synthesis. 

In addition, 24 DAPS were enriched in the pathway of starch and sucrose metabolism, of which 18 DAPS were up-accumulated and only six were down-accumulated in EC (Figure S3, Table 4). In the results of COG analysis, these up-accumulated DAPS were mainly clustered in the classification of “carbohydrate transport and metabolism”. In this pathway, the proteins related with D-Glucose biosynthesis, exoglucanase precursor (3.2.1.21) and endoglucanase 1 precursor (3.2.1.4), were up-accumulated in EC, the fold change reaching 2.07 and 3.941, respectively. In EC of maize inbred line A19, a protein associated with d-glucose biosynthesis, beta d-glucosidase, was also up-accumulated [36]. The accumulated abundance of the proteins mentioned above could promote the synthesis of d-glucose, which is an important source of pyruvate. These results suggested that the high expression of glucose-producing related enzymes in starch and sucrose metabolism might indirectly affect the metabolism of pyruvate from its source.

Glycolysis/gluconeogenesis is a closely related metabolic pathway with starch and sucrose metabolism. Twenty-three DAPS were enriched in this pathway, of which 11 DAPS were up-accumulated and 12 DAPS were down-accumulated in EC (Figure S4, Table 5). Most of the 11 up-accumulated proteins were enzymes and related proteins that catalyze pyruvate production, while the down-accumulated proteins were mainly distributed in the glycolytic pathway of pyruvate to produce acetyl-CoA and to generate ethanol. Accumulation of enzymes involved in pyruvate synthesis were also detected in EC derived from maize inbred lines A19 and H99, respectively [18,36]. Similar to the studies in maize, the content of pyruvate-synthesis related enzymes (i.e., phosphoglycero mutases) also significantly increased during the somatic embryogenesis of *Arabidopsis thaliana* [37]. The main function of phosphoglycero mutases is to catalyze the conversion of 2-phospho-D-glycerate to phosphoenolpyruvate. In our study, we found that the high abundance of heat shock factor-binding protein 1 in EC, which involved in the process of conversion of 2-phospho-D-glycerate to phosphoenolpyruvate catalyzed by phosphoglycero mutases. These results suggested that heat shock factor-binding protein 1 should be involved in the folding of the catalytic process. Meanwhile, pyruvate kinase (2.7.1.40), the key enzyme for pyruvate generation, was also up-accumulated in EC. Overall, the major role of up-accumulated DAPS should be to promote pyruvate formation in EC. One metabolism direction of pyruvate is the formation of lactate. The enzyme (1.1.1.27: l-lactate dehydrogenase A) that catalyzes pyruvate to produce lactate in EC expressed higher than that in NEC, but the expression of various enzymes (i.e., pyruvate decarboxylase 3) that catalyze pyruvate to produce ethanol is lower than that in NEC. The other one metabolism direction of pyruvate is the formation of Acetyl CoA, which is the pivot of energy metabolism and substance metabolism. The abundance of DAPS showed that the expression of various enzymes (i. e., 1.2.4.1: pyruvate dehydrogenase, 1.8.1.4: dihydrolipoyl dehydrogenase and 2.3.1.12: dihydrolipoyl lysine-residue acetyltransferase) in the process of generating acetyl-CoA was lower in EC than that in NEC. All these results indicated that EC mainly relied on not TCA but glycolysis to provide ATP. And the demand for energy and new substances might be lower in EC than in NEC, which also explained why EC usually grew slower than NEC. It is interesting that our results are similar to some stem cell studies, which have proved pyruvate is a very important metabolite for controlling stem cell development. In quiescent state, glycolysis-associated enzymes, such as pyruvate kinase and lactate dehydrogenase A, are highly expressed in pluripotent stem cells. As a result, pyruvate can be catalyzed to produce lactic acid in stem cells. At the same time the synthesis of lipids, proteins or nucleotides are blocked. Stem cells are slow cycling. Along differentiation, energy demands increase and TCA cycle turns on in cells [38,39,40,41,42]. It is suggested that the final destination of pyruvate might determine the direction of callus differentiation during somatic embryogenesis in maize as same as in stem cells. The same regulation mechanism of differentiation might be shared in plant cells and animal cells.

Some lignin substances (i.e., guaiacyl lignin, syringyl lignin and p-Hydroxyphenyl lignin) are generated in phenylpropanoid biosynthesis pathway, in which the DAPS was obviously enriched. Most of DAPS enriched in phenylpropanoid biosynthesis pathway were low abundance in EC (Figure S5). We speculated that this should be an important reason for Y423 EC to have the characteristics of the low lignin content loose structure, and rough surface. 

**Figure 5:**
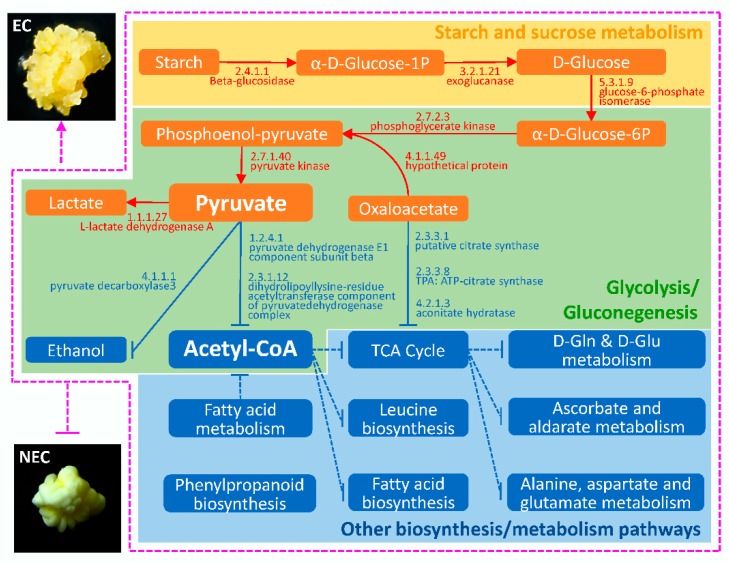
Working model for development of embryogenic and nonembryogenic calli proposed based on DAPS by iTRAQ.

Based on a comprehensive analysis of GO, COG, and KEGG results obtained in this and similar studies, we discovered a new regulation strategy that might affect embryogenic callus development in maize. Pyruvate is the pivot of this model, which involves a lot of biochemical processes such as starch metabolism, glycolysis/gluconeogenesis, pyruvate metabolism, fatty acid biosynthesis and TCA cycle (Figure 5). In maize EC, the up-accumulated DAPS in starch and sucrose metabolism and glycolysis/gluconeogenesis could promote pyruvate generation; meanwhile, the other down-accumulated DAPS could inhibit the conversion of pyruvate to ethanol and to acetyl-CoA. In addition, some DAPS in fatty acid metabolism were also down-accumulated in EC (Figure S6), which further limited the generation of acetyl-CoA. The decrease of acetyl-CoA content would further inhibit leucine biosynthesis, fatty acid biosynthesis, TCA cycle and TCA cycle’s downstream metabolism. These changes could reduce the material accumulation and energy supply, resulting in EC were in a quiescent state. Combined with the reduction of lignin substances, EC could be more likely to maintain in a soft and loose growth state and be more suitable for subculture and differentiation. Conversely, in NEC, more material accumulation and energy supply would lead to rapid growth of calli with high lignin content, hard and smooth surface, not easy to subculture and differentiate.

## 4. Materials and Methods

### 4.1. Plant Materials and Tissue Culture Conditions

The maize elite inbred line Y423 collected by our lab was used in this study, which was the only one of 40 maize inbred lines selected by our lab that can obtain intact somatic embryos [8]. The whole ears were harvested 12–15 days after self-pollination. After husk and sterilization, the immature embryos (size around 2 mm^2^) were inoculated onto the induction medium (100 embryos per plate) in accordance with the procedure of Jimenez and Bangerth [43] with minor modifications as previously described [8]. The immature embryos were incubated in the dark at 24 °C. Buds generated from the immature embryos were removed every other 7 days. After 20–25 days, the primary calli appeared, and then were transferred onto fresh subculture medium. Calli were then subcultured every 20 days on the same subculture medium. After 4–5 times subculturing, the EC and NEC produced, and were selected by histological staining as previously described [8]. We collected around 15 g of EC and NEC separately from 30 plates (derived from one plate of immature embryos) for one biological replicate and then stored them at −80 °C until required. Three biological replicates were performed.

### 4.2. Protein Preparation

EC and NEC samples were ground into powder in liquid nitrogen, extracted with Lysis buffer (7 M Urea, 2 M Thiourea, 4% CHAPS, 40 mM Tris-HCl, pH 8.5) containing 1 mM PMSF and 2 mM EDTA (final concentration). After 5 min, 10 mM DTT (final concentration) was added to the samples. The suspension was sonicated at 200 W for 15 min and then centrifuged at 4 °C, 30,000× *g* for 15 min. The supernatant was mixed well with 5× volume of chilled acetone containing 10% (*v*/*v*) TCA and incubated at −20 °C overnight. After centrifugation at 4 °C, 30,000× *g*, the supernatant was discarded. The precipitate was washed with chilled acetone three times. The pellet was air-dried and dissolved in Lysis buffer (7 M urea, 2 M thiourea, 4% NP40, 20mM Tris-HCl, pH 8.0–8.5). The suspension was sonicated at 200 W for 15 min and centrifuged at 4 °C, 30,000× *g* for 15 min. The supernatant was transferred to another tube. To reduce disulfide bonds in proteins of the supernatant, 10 mM DTT (final concentration) was added and incubated at 56 °C for 1 h. Subsequently, 55 mM IAM (final concentration) was added to block the cysteines, incubated for 1 h in the darkroom. The supernatant was mixed well with 5 5× volume of chilled acetone for 2 h at −20 °C to precipitate proteins. After centrifugation at 4 °C, 30,000× *g*, the supernatant was discarded, and the pellet was air-dried for 5 min, dissolved in 500 μL 0.5 M TEAB (Applied Biosystems, Milan, Italy), and sonicated at 200 W for 15 min. Finally, samples were centrifuged at 4 °C, 30,000× *g* for 15 min. The supernatant was transferred to a new tube and quantified with the Bradford assay using BSA as the calibrant. The proteins in the supernatant were kept at −80 °C for further analysis. 

### 4.3. iTRAQ Labeling and SCX Fractionation

Total protein (100 μg) was taken out of each sample solution and then the protein was digested with Trypsin Gold (Promega, Madison, WI, USA) with the ratio of protein: trypsin = 30:1 at 37 °C for 16 hours. After trypsin digestion, peptides were dried by vacuum centrifugation. Peptides were reconstituted in 0.5M TEAB and processed according to the manufacture’s protocol for 8-plex iTRAQ reagent (Applied Biosystems, Waltham, MA, USA). Briefly, one unit of iTRAQ reagent was thawed and reconstituted in 24 μL isopropanol. The EC replicates were labeled with the iTRAQ tags TP1-113 and TP2-114, and the NEC replicates were labeled with the tags FT1-115 and FT2-116.The peptides were labeled with the isobaric tags, incubated at room temperature for 2 h. The labeled peptide mixtures were then pooled and dried by vacuum centrifugation.

SCX chromatography was performed with a LC-20AB HPLC pump system (Shimadzu, Kyoto, Japan). The iTRAQ-labeled peptide mixtures were reconstituted with 4 mL of buffer A (25 mM NaH_2_PO_4_ in 25% ACN, pH 2.7) and loaded onto a 4.6 × 250 mm Ultremex SCX column containing 5-μm particles (Phenomenex, Torrance, CA, USA). The peptides were eluted at a flow rate of 1 mL/min with a gradient of buffer A for 10 min, 5–60% buffer B (25 mM NaH_2_PO_4_, 1 M KCl in 25% ACN, pH 2.7) for 27 min, 60–100% buffer B for 1 min. The system was then maintained at 100% buffer B for 1 min before equilibrating with buffer A for 10 min prior to the next injection. Elution was monitored by measuring the absorbance at 214 nm, and fractions were collected every 1 min. The eluted peptides were pooled into 20 fractions, desalted with a Strata X C18 column (Phenomenex, Torrance, CA, USA) and vacuum-dried.

### 4.4. LC-ESI-MS/MS Analysis

Each fraction was resuspended in buffer A (5% ACN, 0.1% FA) and centrifuged at 20,000× *g* for 10 min, the final concentration of peptide was about 0.5 μg/μL on average. 10 μL supernatant was loaded on a LC-20AD nanoHPLC (Shimadzu, Kyoto, Japan) by the autosampler onto a 2 cm C18 trap column. Then, the peptides were eluted onto a 10cm analytical C18 column (inner diameter 75 μm) packed in-house. The samples were loaded at 8 μL/min for 4min, then the 35min gradient was run at 300 nL/min starting from 2 to 35% B (95% ACN, 0.1% FA), followed by 5 min linear gradient to 60%, then, followed by 2 min linear gradient to 80%, and maintenance at 80% B for 4 min, and, finally, return to 5% in 1 min. 

Data acquisition was performed with a TripleTOF 5600 System (AB SCIEX, Concord, ON, USA) fitted with a Nanospray III source (AB SCIEX, Concord, ON, USA) and a pulled quartz tip as the emitter (New Objectives, Woburn, MA, USA). Data was acquired using an ion spray voltage of 2.5 kV, curtain gas of 30 psi, nebulizer gas of 15 psi, and an interface heater temperature of 150 °C. The MS was operated with a RP of greater than or equal to 30,000 FWHM for TOF MS scans. For IDA, survey scans were acquired in 250 ms and as many as 30 product ion scans were collected if exceeding a threshold of 120 counts per second (counts/s) and with a 2+ to 5+ charge-state. Total cycle time was fixed to 3.3 s. Q2 transmission window was 100Da for 100%. Four time bins were summed for each scan at a pulser frequency value of 11 kHz through monitoring of the 40 GHz multichannel TDC detector with four-anode channel detect ion. A sweeping collision energy setting of 35 ± 5 eV coupled with iTRAQ adjust rolling collision energy was applied to all precursor ions for collision-induced dissociation. Dynamic exclusion was set for 1/2 of peak width (15 s), and then the precursor was refreshed off the exclusion list. 

### 4.5. Protein Identification and Data Analysis

Raw data files acquired from the Orbitrap were converted into MGF files using Proteome Discoverer 1.2 (PD 1.2, Thermo, SanJose, CA), [5600 msconverter] and the MGF file were searched. Proteins identification were performed by using Mascot search engine (Matrix Science, London, UK; version 2.3.02) against the NCBI *Zea mays* database containing 172,446 sequences (http://www.ncbi.nlm.nih.gov/protein?term=txid4577) with the following parameters: Carbamidomethyl (C), iTRAQ 8 plex (N-term), and Deamidated (NQ) as fixed modification, Gln→pyro-Glu (N-termQ), Oxidation (M), and Deamidated (NQ) as potential variable modifications; the instrument type was set to default; the enzyme was set to trypsin and one max missed cleavages was allowed; a mass tolerance of ±0.05 Da was permitted for intact peptide masses and ±0.1 Da was permitted for fragmented ions. The charge states of peptides were set to +2 and +3. Specifically, an automatic decoy database search was performed in Mascot by choosing the decoy checkbox in which a random sequence of database is generated and tested for raw spectra as well as the real database. To reduce the probability of false peptide identification, only peptides with significance scores (≥20) at the 99% confidence interval by a Mascot probability analysis greater than “identity” were counted as identified. Each confident protein identification involves at least one unique peptide. For protein quantitation, it was required that a protein contains at least two unique peptides. The quantitative protein ratios were weighted and normalized by the median ratio in Mascot. We only used ratios with *p*-values < 0.05, and only fold changes of >1.2 were considered as significant. 

### 4.6. Bioinformatics Analysis 

Functional annotations of differentially accumulated protein species were performed using Gene Ontology (http://www.geneontology.org). The Clusters of Orthologous Groups of proteins (COG) (http://www.ncbi.nlm.nih.gov/COG/) database was carried out for functional classification of DAPS, and the Kyoto Encyclopedia of Genes and Genomes (KEGG) (http://www.genome.jp/kegg/ or http://www.kegg.jp/) was used to predict the main metabolic pathways and biochemical signals transduction pathways that involved the DAPS. A *p*-value ≤ 0.05 was used as the threshold to determine the significant enrichments of GO and KEGG pathways. 

### 4.7. Quantitative Real-Time PCR Analysis

Total RNA extracted from the EC and NEC was treated with DNase I to remove genomic DNA. The first strand cDNA synthesis and the qRT-PCR were carried out using the SuperScript™ III First-Strand Synthesis SuperMix (Invitrogen, Carlsbad, CA, USA) and the SYBR Green JumpStart™ Taq ReadyMix™ (Sigma-Aldrich, St. Louis, MO, USA), respectively. qRT-PCR was carried out in the ABI PRISM 7500 sequence detection system (Waltham, MA, USA) according to the manufacturer’s instructions. To validate the DAPS obtained from iTRAQ, 12 genes were subjected to quantitative real-time PCR. Each 20 ml reaction mixture contained 10 mM each primers 0.4 ml, 2× SYBR Premix Ex Taq 10 ml, 50× ROX Reference Dye II 0.4 mL and cDNA (1:5) template 2 ml. Gene-specific primers were designed using Primer Express 3.0 (Waltham, MA, USA), and amplified maize *actin* 1 and *gapdh* were used as reference genes (Supplementary Table S1). Three replications were performed for each sample.

## 5. Conclusions

We used the iTRAQ technique to perform quantitative proteome analysis of EC and NEC derived from Y423 inbred maize line. This approach identified some new proteins involved in pyruvate metabolism (i.e., pyruvate dehydrogenase E1 component subunit beta and dihydrolipoyllysine-residue acetyltransferase component of pyruvate dehydrogenase complex), TCA cycle (i.e., citrate synthase and aconitate hydratase), fatty acid metabolism (i.e., acetyl-CoA acetyltransferase), and phenylpropanoid biosynthesis (i.e., peroxidase 39 isoform and 1-Cys peroxiredoxin) that were not previously known to be associated with EC and SE developing. Based on functional analysis, we proposed a regulation strategy for reprograming of somatic cell to EC in maize. This strategy suggests that accumulation of pyruvate and the alterations in abundances of proteins in several associated pathways (i.e., starch and sucrose metabolism and glycolysis/gluconeogenesis) might be responsible for differences between EC and NEC. In summary, our analysis of differences in the protein profiles between EC and NEC increased our understanding of the mechanisms of EC development in maize.

## Figures and Tables

**Table 1:** Protein information in the COG classification of including only the upregulated DAPS.

Protein Accession	Fold Change	Description	COG Code
gi|195605264|gb|ACG24462.1|	1.311	Histone H2A [*Zea mays*]	B
gi|195635409|gb|ACG37173.1|	1.314	Histone H4 [*Zea mays*]	B
gi|162458988|ref|NP_001105357.1|	1.41	Histone H2A [*Zea mays*]	B
gi|195618876|gb|ACG31268.1|	1.467	Histone H2A [*Zea mays*]	B
gi|195616386|gb|ACG30023.1|	1.541	Histone H2A [*Zea mays*]	B
gi|195617710|gb|ACG30685.1|	1.906	Histone H2A [*Zea mays*]	B
gi|194693822|gb|ACF80995.1|	2.084	Unknown [*Zea mays*]	B
gi|226496461|ref|NP_001140401.1|	1.234	Calmodulin [*Zea mays*]	DTZR
gi|226501230|ref|NP_001149695.1|	1.29	Cell division protein ftsz [*Zea mays*]	D
gi|226494524|ref|NP_001150546.1|	1.321	Serine/threonine-protein phosphatase 2A activator 2 [*Zea mays*]	DT
gi|293331121|ref|NP_001167965.1|	1.334	Uncharacterized protein LOC100381681 [*Zea mays*]	D
gi|212276110|ref|NP_001130839.1|	1.315	Uncharacterized protein LOC100191943 [*Zea mays*]	K
gi|226506726|ref|NP_001141599.1|	1.372	Uncharacterized protein LOC100273717 [*Zea mays*]	KLJ
gi|226490952|ref|NP_001152536.1|	1.412	ATP-dependent RNA helicase DDX23 [*Zea mays*]	KLJ
gi|226496545|ref|NP_001148353.1|	1.53	Small nuclear ribonucleoprotein Sm D1 [*Zea mays*]	K
gi|195656155|gb|ACG47545.1|	1.565	Small nuclear ribonucleoprotein Sm D3 [*Zea mays*]	K
gi|219363641|ref|NP_001136911.1|	1.586	Uncharacterized protein LOC100217069 [*Zea mays*]	K
gi|226503431|ref|NP_001140836.1|	1.647	Uncharacterized protein LOC100272912 [*Zea mays*]	K
gi|194699902|gb|ACF84035.1|	2.544	U6 snrna-associated Sm-like protein lsm3 [*Zea mays*]	K

To further investigate biological functions of these proteins, 500 DAPS (79.1%) were mapped to 106 pathways in the KEGG database, in which 33 KEGG pathways were significantly enriched (*p*-value ≤ 0.05) (Supplementary Table S3). “Metabolic pathways” (*p* = 1.19 × 10^−11^) was the most represented pathway, followed by “biosynthesis of secondary metabolites” (*p* = 4.09 × 10^−10^) and “pyruvate metabolism” (*p* = 8.74 × 10^−5^). Some DAPS were also significantly enriched in pathways of “starch and sucrose metabolism” (*p* = 0.001436749), “Fatty acid metabolism” (*p* = 0.006471598), “glycolysis/gluconeogenesis” (*p* = 0.01189691), and so on.

**Table 2:** Candidate genes for qRT-PCR.

Code	Protein Accession	Fold Change	Description
UP76	gi|195614828|gb|ACG29244.1|	4.26	Nucleotide pyrophosphatase/phosphodiesterase [*Zea mays*]
UP12	gi|195621264|gb|ACG32462.1|	4.46	histone H1 [*Zea mays*]
UP250	gi|414878739|tpg|DAA55870.1|	4.272	TPA: putative O-Glycosyl hydrolase superfamily protein [*Zea mays*]
UP322	gi|413952293|gb|AFW84942.1|	4.218	Putative subtilase family protein [*Zea mays*]
UP316	gi|414866956|tpg|DAA45513.1|	4.841	Peroxidase superfamily protein [*Arabidopsis thaliana*]
UP108	gi|414586772|tpg|DAA37343.1|	6.499	Polygalacturonase inhibiting protein 1 [*Arabidopsis thaliana*]
UP292	gi|195654029|gb|ACG46482.1|	6.87	Rhicadhesin receptor precursor [*Zea mays*]
UP283	gi|1352469|sp|P49174.1|INVA_MAIZE	4.771	Cell-wall invertase 2 [Arabidopsis thaliana]
DOWN60	gi|257626267|emb|CBD24252.1|	0.152	Unnamed protein product [*Zea mays*]
DOWN90	gi|239050557|ref|NP_001132867.2|	0.233	S-adenosylmethionine synthetase family protein [*Arabidopsis thaliana*]
DOWN205	gi|226500748|ref|NP_001140439.1|	0.199	NAD(P)-binding Rossmann-fold superfamily protein [*Arabidopsis thaliana*]
DOWN1	gi|162458192|ref|NP_001105950.1|	0.156	Terpenoid cyclases/Protein prenyltransferases superfamily protein [*Arabidopsis thaliana*]

**Table 3:** Information of DAPS in ribosome pathway.

Protein Accession	Enzyme Commission	Fold Change	Regulated	Description
gi|212722264|ref|NP_001132360.1|	S12e	1.436	UP	40S ribosomal protein S12 isoform 1 [*Zea mays*]
gi|195605820|gb|ACG24740.1|	S20e	1.352	UP	40S ribosomal protein S20 [*Zea mays*]
gi|195623210|gb|ACG33435.1|	S20e	1.247	UP	40S ribosomal protein S20 [*Zea mays*]
gi|162463985|ref|NP_001105635.1|	S24e	1.245	UP	40S ribosomal protein S24 [*Zea mays*]
gi|226532924|ref|NP_001146968.1|	S24e	0.833	DOWN	40S ribosomal protein S24 [*Zea mays*]
gi|195634815|gb|ACG36876.1|	S29e	2.384	UP	40S ribosomal protein S29 [*Zea mays*]
gi|226499664|ref|NP_001149323.1|	S30e	1.394	UP	40S ribosomal protein S30 [*Zea mays*]
gi|162464000|ref|NP_001105390.1|	LP1, LP2	0.735	DOWN	60S acidic ribosomal protein P2B [*Zea mays*]
gi|212275159|ref|NP_001130808.1|	L13Ae	1.46	UP	60S ribosomal protein L13a [*Zea mays*]
gi|195623288|gb|ACG33474.1|	L13Ae	1.206	UP	60S ribosomal protein L13a [*Zea mays*]
gi|195642172|gb|ACG40554.1|	L17e	1.536	UP	60S ribosomal protein L17 [*Zea mays*]
gi|195656195|gb|ACG47565.1|	L17e	1.336	UP	60S ribosomal protein L17 [*Zea mays*]
gi|195621692|gb|ACG32676.1|	L24e	1.342	UP	60S ribosomal protein L24 [*Zea mays*]
gi|195646398|gb|ACG42667.1|	L28e	1.39	UP	60S ribosomal protein L28 [*Zea mays*]
gi|195625790|gb|ACG34725.1|	L35e	1.535	UP	60S ribosomal protein L35 [*Zea mays*]
gi|413922853|gb|AFW62785.1|	L6e	1.362	UP	60S ribosomal protein L6 [*Zea mays*]
gi|195656241|gb|ACG47588.1|	L7Ae	1.352	UP	60S ribosomal protein L7a [*Zea mays*]
gi|212274675|ref|NP_001130101.1|	L10e	1.791	UP	Acid phosphatase 1 [*Zea mays*]
gi|195642478|gb|ACG40707.1|	S19	1.24	UP	Glycine-rich RNA-binding protein 2 [*Zea mays*]
gi|212722104|ref|NP_001132260.1|	L11e	1.24	UP	Hypothetical protein [*Zea mays*]
gi|226509268|ref|NP_001141489.1|	LP0	0.798	DOWN	Hypothetical protein [*Zea mays*]
gi|162460468|ref|NP_001105280.1|	L31e	1.643	UP	Putative 60S ribosomal protein L31 [*Zea mays*]
gi|132668| sp|P08529.2|RK14_MAIZE	L14	0.709	DOWN	Recname: Full=50S ribosomal protein L14, chloroplastic
gi|3264605|gb|AAC24573.1|	L23Ae	1.39	UP	Ribosomal protein L25 [*Zea mays*]
gi|226497590|ref|NP_001148801.1|	L7Ae	1.312	UP	TPA: 60S ribosomal protein L7a [*Zea mays*]
gi|414873848|tpg|DAA52405.1|	S4e	1.475	UP	TPA: ribosomal protein S4 [*Zea mays*]
gi|212721318|ref|NP_001131538.1|	L18e	1.414	UP	Uncharacterized protein LOC100192878 [*Zea mays*]
gi|224030849|gb|ACN34500.1|	L6e	1.793	UP	Unknown [*Zea mays*]
gi|342591|gb|AAA84482.1|	RP-L16	0.768	DOWN	Unknown protein (chloroplast) [*Zea mays*]
gi|257722902|emb|CBD23964.1|	L19e	1.268	UP	Unnamed protein product [*Zea mays* subsp. Mays]

**Table 4:** Information of DAPS in starch and sucrose metabolism pathway.

Protein Accession	Enzyme Commission	Fold Change	Regulated	Description
gi|162462658|ref|NP_001105539.1|	3.2.1.1	2.29	UP	Alpha-amylase precursor [*Zea mays*]
gi|226530773|ref|NP_001150278.1|	3.2.1.1	1.627	UP	Alpha-amylase precursor [*Zea mays*]
gi|1352469|sp|P49174.1|INVA_MAIZE	3.2.1.26	4.771	UP	Beta-fructofuranosidase/cell wall isozyme;
gi|226495335|ref|NP_001151458.1|	3.2.1.4	3.941	UP	Endoglucanase 1 precursor [*Zea mays*]
gi|8809764|gb|AAF79936.1|	3.2.1.21	2.07	UP	Exoglucanase precursor [*Zea mays*]
gi|195639126|gb|ACG39031.1|	2.7.1.4	1.261	UP	Fructokinase-2 [*Zea mays*]
gi|162460322|ref|NP_001105368.1|	5.3.1.9	1.324	UP	Glucose-6-phosphate isomerase, cytosolic [*Zea mays*]
gi|308080756|ref|NP_001183080.1|	3.2.1.15	1.683	UP	Hypothetical protein precursor [*Zea mays*]
gi|413933069|gb|AFW67620.1|	3.2.1.21	3.115	UP	Hypothetical protein ZEAMMB73_549956 [*Zea mays*]
gi|413922001|gb|AFW61933.1|	2.4.1.14	1.242	UP	Putative sucrose-phosphate synthase family protein [*Zea mays*]
gi|413952450|gb|AFW85099.1|	3.1.3.12/ 2.4.1.15	1.325	UP	Putative trehalose phosphatase/synthase family protein [*Zea mays*]
gi|194740442|gb|ACF94692.1|	2.4.1.1	1.228	UP	Starch phosphorylase 1 precursor [*Zea mays*]
gi|525344616|ref|NP_001266691.1|	2.4.1.13	1.203	UP	Sucrose synthase 1 [*Zea mays*]
gi|414867410|tpg|DAA45967.1|	3.2.1.28	2.643	UP	TPA: hypothetical protein ZEAMMB73_076801 [*Zea mays*]
gi|414879406|tpg|DAA56537.1|	3.1.1.11	2.287	UP	TPA: pectinesterase [*Zea mays*]
gi|414885330|tpg|DAA61344.1|	3.1.3.12/ 2.4.1.15	1.84	UP	TPA: putative trehalose phosphatase/synthase family protein [*Zea mays*]
gi|257713456|emb|CBD15747.1|	3.2.1.37	1.917	UP	Unnamed protein product [*Zea mays*]
gi|257713456|emb|CBD15747.1|	3.2.1.21	1.917	UP	Unnamed protein product [*Zea mays*]
gi|413946674|gb|AFW79323.1|	2.7.7.27	0.553	DOWN	ADP-glucose pyrophosphorylase large subunit [*Zea mays*]
gi|413920595|gb|AFW60527.1|	2.4.1.14	0.55	DOWN	Putative sucrose-phosphate synthase family protein [*Zea mays*]
gi|1351136|sp|P49036.1|SUS2_MAIZE	2.4.1.13	0.808	DOWN	Sucrose-UDP glucosyltransferase 2
gi|226529913|ref|NP_001146685.1|	3.1.1.11	0.773	DOWN	TPA: pectinesterase [*Zea mays*]
gi|414865602|tpg|DAA44159.1|	3.1.3.12/ 2.4.1.15	0.728	DOWN	TPA: putative trehalose phosphatase/synthase family protein [*Zea mays*]
gi|226505764|ref|NP_001149225.1|	1.1.1.22	0.602	DOWN	UDP-glucose 6-dehydrogenase [*Zea mays*]

**Table 5:** Information of DAPS in glycolysis /gluconeogenesis pathway.

Protein Accession	Enzyme Commission	Fold Change	Regulated	Description
gi|6225010|sp|P93629.1|ADHX_MAIZE	1.1.1.1	1.386	UP	Alcohol dehydrogenase class-III;
gi|195613242|gb|ACG28451.1|	5.1.3.3	1.7	UP	Aldose 1-epimerase precursor [*Zea mays*]
gi|162460322|ref|NP_001105368.1|	5.3.1.9	1.324	UP	Glucose-6-phosphate isomerase, cytosolic [*Zea mays*]
gi|162457962|ref|NP_001105090.1|	4.2.1.11	1.58	UP	Heat shock factor-binding protein 1 [*Zea mays*]
gi|226528284|ref|NP_001150088.1|	4.2.1.11	1.609	UP	Heat shock factor-binding protein 1 [*Zea mays*]
gi|413956284|gb|AFW88933.1|	4.1.1.49	1.316	UP	Hypothetical protein ZEAMMB73_030639 [*Zea mays*]
gi|195621388|gb|ACG32524.1|	1.1.1.27	1.774	UP	L-lactate dehydrogenase A [*Zea mays*]
gi|226509797|ref|NP_001142404.1|	2.7.2.3	1.334	UP	Phosphoglycerate kinase isoform 3 [*Zea mays*]
gi|195620854|gb|ACG32257.1|	2.7.1.40	1.712	UP	Pyruvate kinase, cytosolic isozyme [*Zea mays*]
gi|226528689|ref|NP_001141131.1|	1.1.1.1	1.288	UP	TPA: putative alcohol dehydrogenase superfamily protein [*Zea mays*]
gi|291047868|emb|CBK51447.1|	1.2.1.3	1.272	UP	Unnamed protein product [*Zea mays*]
gi|195614676|gb|ACG29168.1|	1.2.1.3	0.629	DOWN	Aldehyde dehydrogenase, dimeric NADP-preferring [*Zea mays*]
gi|162460054|ref|NP_001105576.1|	1.2.1.3	0.576	DOWN	Aldehyde dehydrogenase2 [*Zea mays*]
gi|363543269|ref|NP_001241850.1|	1.1.1.2	0.3	DOWN	Aldose reductase [*Zea mays*]
gi|293335591|ref|NP_001169718.1|	1.8.1.4	0.766	DOWN	Dihydrolipoyl dehydrogenase [*Zea mays*]
gi|226499350|ref|NP_001142314.1|	2.3.1.12	0.8	DOWN	Dihydrolipoyl lysine-residue acetyltransferase component of pyruvate dehydrogenase complex [*Zea mays*]
gi|120670|sp|P08735.2|G3PC1_MAIZE	1.2.1.12	0.69	DOWN	Glyceraldehyde-3-phosphate dehydrogenase 1, cytosolic
gi|226531804|ref|NP_001148246.1|	1.1.1.2	0.533	DOWN	NAD(P)H-dependent oxidoreductase [*Zea mays*]
gi|413937078|gb|AFW71629.1|	6.2.1.1	0.674	DOWN	Putative AMP-dependent synthetase and ligase superfamily protein [*Zea mays*]
gi|162457852|ref|NP_001105052.1|	4.1.1.1	0.777	DOWN	Pyruvate decarboxylase 3 [*Zea mays*]
gi|226529151|ref|NP_001150473.1|	1.2.4.1	0.588	DOWN	Pyruvate dehydrogenase E1 component subunit beta [*Zea mays*]
gi|226528639|ref|NP_001140759.1|	1.2.4.1	0.613	DOWN	Uncharacterized protein LOC100272834 [*Zea mays*]
gi|219887835|gb|ACL54292.1|	1.2.1.12	0.832	DOWN	Unknown [*Zea mays*]

## Supplementary Materials

Supplementary materials can be found at http://www.mdpi.com/1422-0067/19/12/4004/s1.

## Abbreviations

COG	clusters of orthologous groups of proteins
DAPS	differential abundance protein species
EC	embryogenic calli
FDR	false discovery rate
GM	genetically modified
GO	gene ontology
iTRAQ	isobaric tags for relative and absolute quantification
NEC	nonembryogenic calli
SE	Somatic embryo
TCA	tricarboxylic acid
UDP	uracil-diphosphate
