# Supplementary material for: iTRAQ-Based Quantitative Proteomic Analysis of Embryogenic and Non-embryogenic Calli Derived from a Maize (Zea mays L.) Inbred Line Y423
Source: Int J Mol Sci. 2018 Dec 12;19(12):4004. doi: 10.3390/ijms19124004 (PMC6321184; doi:10.3390/ijms19124004)
Supplement: Supplementary file 1 [file ijms-19-04004-s001.zip › Table S1 Primers of qRT.pdf]

Table S1 Primers of qRT-PCR

TDFs	RT-PCR forward primer(5′-3′)	RT-PCR reverse primer(5′-3′)
UP76	AAGGCTCCAGTCTACCCACG	AGCGTTGTTTGGGGCAAG
UP12	TAGCAGCGTCGTAGTCGTGAT	TGGCTCATCATCTTCGGCT
UP250	CGGAAATACAACCAGGGACTCA	CTTGTGGAGGAGCGGTGCA
UP322	TGGAGTTCACTTGGATTGGATTC	AGCGAACATACAGCAACCCAG
UP316	GCTTCGTGCGGGCTCAA	CCCAACAACCCCAGCCT
UP108	TCTTTCCTGAACCTGAGCCAC	ACAACGACCTCTGCGGGA
UP292	CCGCTTTCCCCGTGGTT	GGAGTGCCGCTTCCCTGAT
UP283	CCCAACGCTCCGCTGTA	CGGCTTCTCCCACTCGC
DOWN60	CTGCTGGGTGGACATCGCT	TTCCAGACCCCAGCAAGCC
DOWN90	CGGACGCCGTGCTTGAC	CCAGGGCCACATGTTCGG
DOWN1	GATGGCTGCCCCAACAC	AGCGACTAAATCTGGACTACCA
DOWN205	TCTTCCTCGCCAGCAACC	GGACACGACAGCAAAACAGC
Actin1	CGATTGAGCATGGCATTGTCA	CCCACTAGCGTACAACGAA
α-tubulin	ACTTCATGCTTTCGTCCTACGCTCCA	CTGGGAGGCTGGTAGTTGATTC
