# Supplementary figures and images for: iTRAQ-Based Quantitative Proteomic Analysis of Embryogenic and Non-embryogenic Calli Derived from a Maize (Zea mays L.) Inbred Line Y423
Source: Int J Mol Sci. 2018 Dec 12;19(12):4004. doi: 10.3390/ijms19124004 (PMC6321184; doi:10.3390/ijms19124004)

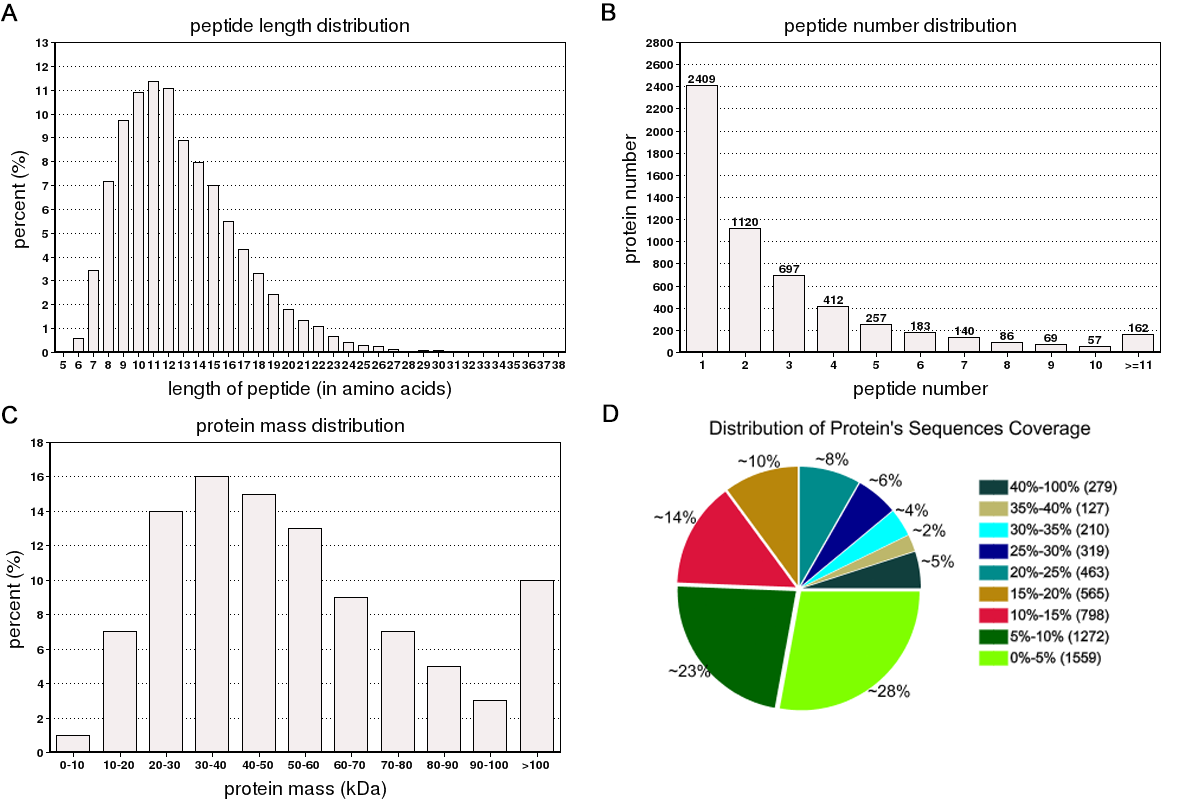

Supplement: Supplementary file 1 [file ijms-19-04004-s001.zip › Fig S1.tif]
